# Development of a program theory for clinical pathways in hospitals: protocol for a realist review

**DOI:** 10.1186/s13643-019-1046-0

**Published:** 2019-06-08

**Authors:** Adegboyega K. Lawal, Gary Groot, Donna Goodridge, Shannon Scott, Leigh Kinsman

**Affiliations:** 1 College of Pharmacy and Nutrition, University of Saskatchewan, Saskatoon, Canada; 2 College of Medicine, University of Saskatchewan, Saskatoon, Canada; 3 Department of Medicine, College of Medicine, University of Saskatchewan, Saskatoon, Canada; 4 Faculty of Nursing, University of Alberta Edmonton, Edmonton, Canada; 5 University of Newcastle and Mid-North Coast Local Health District, Port Macquarie Base Hospital, Port Macquarie, Australia

**Keywords:** Clinical pathways, Realist review, Hospitals, Mechanisms, Implementation, Evaluation, Theory

## Abstract

**Background:**

Despite the increased utilization of clinical pathways (CPWs) as a strategy to improve patient and system outcomes in hospitals, there remain ongoing challenges with their conceptualization, implementation, and evaluation. Theories that explain how CPWs work in hospitals are lacking, making it difficult to identify important factors for sustaining changes arising from CPWs implemented in hospitals. The objective of this realist review is to develop a program theory for CPWs in hospitals.

**Methods:**

This is a protocol for a realist review. The review will use a six-step iterative process to develop a program theory for CPWs in hospitals: (1) development of a preliminary program theory; (2) search strategy and literature search; (3) study selection and appraisal; (4) data extraction; (5) data analysis and synthesis; and (6) stakeholder engagement. In addition to searching the gray literature and contacting authors, we will search electronic databases such as MEDLINE, NHSEED, CINAHL EBSCO, HMIC, and PsycINFO. Studies will be included based on their ability to provide data that test some aspect of the program theory. Two independent reviewers will select, screen, and extract data related to the program theory from all relevant sources. A realist logic of analysis will be used to identify all context-mechanism-outcome heuristics that explains how CPWs implemented in hospitals translates to better health system outcomes.

**Discussion:**

Overall, the review aims to develop a program theory for CPWs in hospitals and to explore how, why, to what extent, and in what contexts does the implementation of CPWs in hospitals contribute to better health system outcomes. As a result, the review will provide a theoretical framework of how CPWs work in hospitals.

**Systematic review registration:**

PROSPERO CRD42018103220

**Electronic supplementary material:**

The online version of this article (10.1186/s13643-019-1046-0) contains supplementary material, which is available to authorized users.

## Background

In this era of increasingly scarce resources, administrators and healthcare providers are tasked continually with the responsibility of providing high-quality patient-centered care [1, 2]. Hospitals play a vital role in patient management by providing acute and specialist services, which constitutes a core feature of well-functioning health systems [3]. Many health systems have turned to new ways of providing patient care to maximize clinical efficiency [4]. Robust quality improvement initiatives to improve the quality of patient care have been trialed across different health systems to enhance the quality of the care delivered [4]. Examples of these initiatives, such as Six Sigma and Lean management, have been implemented in various hospital contexts [4].

Clinical pathways (CPWs), also known as care maps, algorithms or protocols are evidence-based multidisciplinary care plans that detail a stepwise approach to the management of patients with a specific disease [5, 6]. CPWs are used to translate universal clinical guidelines into local protocols to influence clinical practice [7]. Originating in the USA in the 1980s [8], CPWs have been implemented across many health systems and are primarily used in hospitals to reduce clinical variation by standardizing care processes, promoting interprofessional teamwork, containing costs and improving patient outcomes [9, 10]. CPWs are commonly implemented as web-based tools to support clinical decision-making processes. Hence, it is timely to develop a testable theory that can explain how, why, to what extent and in what contexts effective implementation of CPWs in hospitals contributes to better health system outcomes.

Despite the widespread use of CPWs in healthcare settings, evidence on their effectiveness in hospital settings has been equivocal [11, 12] A Cochrane systematic review on effects of clinical pathways in hospitals by Rotter et al. (2010) concluded that CPWs have the potential to reduce in-hospital complications and improve documentation among healthcare providers, although there was no evidence of differences in hospital readmissions or mortality rates [11]. Conversely, Doig et al. (2008) and Process et al. (2014) reported increased mortality within a period of 90 days after using CPWs to manage stroke and septic shock patients [13, 14]. A systematic review by Deneckere et al. (2012) on the effects of CPWs on teamwork found little evidence of improvement in staff knowledge, interprofessional documentation, team communication, or team relations [10].

The conceptualization, implementation, and evaluation of CPWs in healthcare settings are an on-going challenge for program managers and CPWs researchers [14, 15]. Several challenges plaguing this area of investigation include the lack of a common definition for CPWs in healthcare, scarcity of evidence-based strategies for effective implementation and the use of weak evaluative designs [11]. One of the common challenges facing CPWs in hospitals is the lack of explicit theories on how CPWs work in these practice settings [16]. Hospitals are complex healthcare environments with diverse stakeholders and structures interwoven to achieve different and common goals [17]. Adoption of interventions with multifaceted components (complex interventions), such as CPWs in hospitals, require a broader perspective and understanding of the hierarchical layers within the hospital environment [18, 19].

The use of theory in implementing evidence-based interventions is vital to the operationalization, adoption, and replication of new knowledge in hospitals. Additionally, interventions underpinned by the theory are more likely to provide meaningful information on the sequence of events and how they relate to the intended program outcomes [18]. This information can, in turn, be used to improve the overall program or adapted for a different setting. Apart from the lack of a program theory for CPWs in hospitals, our rationale for conducting a realist review stems from the methodological constraints experienced by our review team during the conduct of the Cochrane systematic review update (Rotter and colleagues: Clinical Pathways for secondary care and the effect on professional practice, patient outcomes, length of stay and hospital costs, unpublished) on effects of CPWs in hospitals. The systematic review update, like the first version [11], was limited in explaining how hospital-based CPWs work or influence outcomes from a health system perspective. This limitation may be due to the inability of systematic reviews to fully capture the reality and complexities associated with the professional healthcare environment.

Realist philosophy acknowledges that the world is “real” and humans interact with reality which limits or constructs our interpretation [20]. Realist reviews aim to examine “what works for whom under what circumstances, how and why.” Originally developed by Ray Pawson, a realist review utilizes multiples sources of information and relevant study designs linked to the program theory to explain the causal relationships between the contexts, mechanisms, and outcomes with the goal of refining the program theory [21]. A program theory is an expression of how of an intervention or its components lead to intended outcomes [22]. The program theory seeks to provide decision-makers with an in-depth understanding of an intervention and how it does or does not work in different contexts, thus making it appropriate for evaluating complex interventions such as CPWs. This notion is important in our review because there is a likelihood of variation in the level of understanding or purpose of a CPW for different groups of care providers within the same hospital. This makes a realist synthesis a suitable approach to explore and understand the complexities associated with how clinical pathways influence health system outcomes.

While contexts are conditions that an intervention operates in (not necessarily settings), mechanisms in simple terms are “what cause things to happen” [21, 23]. A mechanism is a generative force that leads to an intended or unintended outcome triggered by one or more contextual factors [24, 25]. Mechanisms are usually latent and often operationalized as the interaction between resources and context to generate an outcome [26]. Thus, realists assert that the interaction between the context(s) and mechanisms can shed light on the patterns of outcomes observed. This theory-based synthesis will utilize a context-mechanism-outcome (CMO) configuration to develop a program theory for implementing clinical pathways in hospitals [21]. CMO configurations will permit the formation of testable hypotheses that can be used for confirmation or refinement of an initial program theory [27]. For example, the identification of a hypothesis for implementing CPWs in hospitals that value “interprofessional collaboration” may provide valuable information that can be used to confirm or refute this aspect of the program theory. The proposed realist review will permit the development of testable hypotheses that can improve our understanding of CPWs in healthcare and how they contribute to better health system outcomes.

The objective of this realist review is to develop a program theory for CPWs work in hospitals.

The primary review question is: “How, why, to what extent and in what contexts does the implementation of clinical pathways in hospitals translate to better health system outcomes?” To adequately capture the breadth of available evidence and to allow for comparison with our ongoing systematic review on the effects on CPWs in hospitals, we will focus on the following health system outcomes: length of hospital stay, costs, in-hospital mortality, adherence to recommended practice, and in-hospital complications.

The secondary review questions are as follows:

I. What contexts facilitate or hinder the implementation of clinical pathways in hospitals?
II. What mechanisms support the implementation of clinical pathways in hospitals?
III. Does the program theory for clinical pathways in hospitals fare better with some group of individuals, interpersonal relations, institutions, and infrastructures?
IV. Are there differences in the understanding by different groups of what hospital clinical pathways are trying to accomplish?

## Methods

**Fig. 1:**
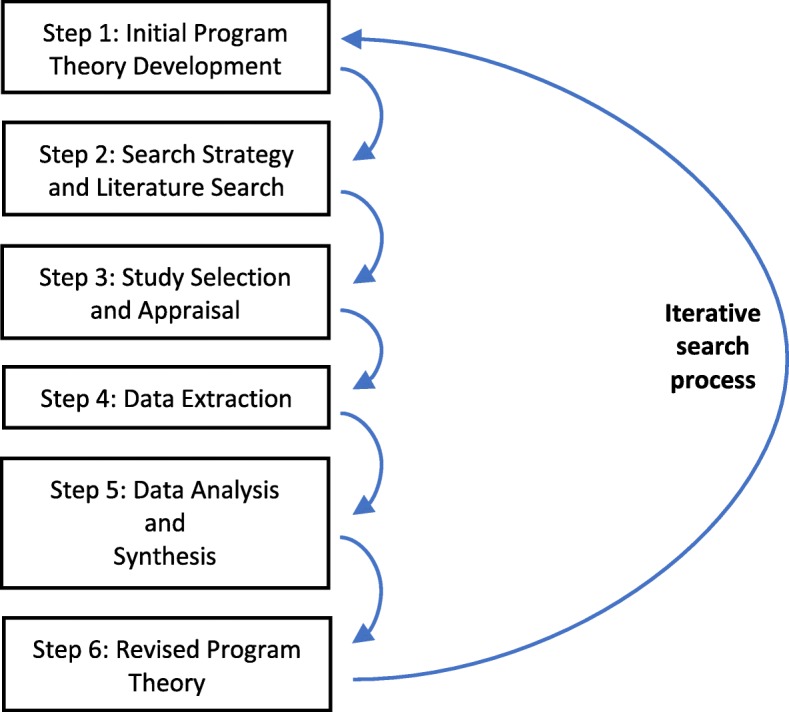
Steps to conduct a realist review. Adapted from Molnar and colleagues

The present protocol has been registered with the PROSPERO database (registration number: CRD42018103220) and is being reported in accordance with the reporting guidance the Preferred Reporting Items for Systematic Reviews and Meta-Analyses Protocols (PRISMA-P) statement [28] (See PRISMA-P checklist in Additional file 1). With content and clinical expertise and librarian support, our team will review the CPWs literature with the aim of developing a program theory. We will adopt the approach by Molnar et al. (2015) [29], initially designed by Pawson [23] for conducting realist review, to develop a program theory for CPWs in hospitals. The steps involve (1) development of a preliminary program theory; (2) search strategy and literature search; (3) study selection and appraisal; (4) data extraction; (5) data analysis and synthesis; and (6) stakeholder engagement (Fig. 1).

This review will be reported following the Realist and Meta-Review Evidence Synthesis: Evolving Standards (RAMESES) reporting guidelines for realist synthesis [27]. For this review, we will adopt the working definition for a CPW originally developed by Rotter and colleagues [30] and refined by Lawal and colleagues [6]: (1) the intervention was a structured multidisciplinary plan of care; (2) the intervention was used to translate guidelines or evidence into local structures; (3) the intervention detailed the steps in a course of treatment or care in a plan, pathway, algorithm, guideline, protocol, or other ‘inventory of actions’ (i.e., the intervention had time-frames or criteria-based progression); and (4) the intervention aimed to standardize care for a specific population. An intervention meeting all four criteria will be identified as a CPW.

### Initial program theory development

We choose a realist approach to develop a program theory for CPWs in hospitals due to the heterogeneous nature of the evidence base generated by our ongoing systematic review (Rotter and colleagues: Clinical Pathways for secondary care and the effect on professional practice, patient outcomes, length of stay and hospital costs, unpublished) on the effects of clinical pathways in hospitals. The initial program theory will be derived from the preliminary findings of our on-going Cochrane systematic review update on the effects of CPWs in hospitals. International participants outside Saskatchewan will be involved throughout the theory building phase. We will identify and group the processes, strategies, resources, and outcomes resulting from the implementation of CPWs in hospitals based on the objective and theoretical accounts of the authors. The review team will construct the preliminary program theory by generating hypotheses of “how,” “to what extent,” and “in what contexts” does the implementation of clinical pathways in hospitals contribute to better health system outcomes. This process will be carried out in close consultation with a realist methodologist, Dr. Gill Westhorp. With support from the Saskatchewan Ministry of Health and the Saskatchewan Health Quality Council’s Variation and Appropriateness Working Group (VAWG) and our international stakeholders, we will consult with a group of multidisciplinary content experts including physicians, nurses, researchers, project managers, and other relevant frontline users of CPWs based in and outside Saskatchewan, to provide critical insights to refine the program theory.

### Search strategy and literature search

To develop an initial understanding of how CPWs in hospitals are intended to translate to better health system outcomes, we will conduct a focused examination of the CPWs literature. As recommended by the RAMESES guidelines, our search strategy and search terms will be guided by hypotheses developed from the preliminary program theory [27]. We will start with the initial search terms identified from the on-going systematic review update of CPWs effects in hospitals. Search terms will include: “clinical pathways,” “care pathways,” “critical pathways,” “clinical protocol,” “implementation,” “effective,” “hospital,” “patient,” “physician(s),” “treatment,” “theory,” “models,” “strategy(s).” These search terms will be examined by the review team in consultation with an information scientist and expanded as necessary. The search process will commence with Medline and Google scholar and be further expanded to other databases such as NHSEED, CINAHL EBSCO, HMIC, and PsycINFO. All sources of information related to our review objectives will be considered. We will assess saturation iteratively by asking after each stage or cycle of searching whether the latest information has added anything new to our understanding of the intervention and whether further searching is likely to add new knowledge [21]. Since CPWs emerged in the 1980s [8], we will search for published and unpublished studies from 1980 to present with no language restrictions. See (Additional file 2, part A) for an example of the initial MEDLINE search strategy used for the on-going Cochrane systematic review on the effects of CPWs in hospitals.

Additionally, we will search gray literature and websites of organizations involved with the implementation of CPWs, such as Intermountain Healthcare [31], European Pathway Association [32], The National Institute for Health and Care Excellence [33], etc. We will consult with authors of included primary articles for other relevant publications. We will use a snowball sampling method [34] to examine references of retained articles during the initial screening phase. We will follow an iterative approach for searching the literature to refine or refute the initial program theory. Overall, the initial development and refinement of the search strategy and conducting the literature search will follow an iterative process.

### Study selection and appraisal

Iteratively, we will use a two-step process to screen all potentially eligible retrieved studies beginning with the abstracts, followed by full-text assessment of the included abstracts. Two review members will independently screen all retrieved studies from the search process using the following inclusion criteria: (1) intervention meets the following definition for a clinical pathway (i) the intervention was a structured multidisciplinary plan of care; (ii) the intervention was used to translate guidelines or evidence into local structures; (iii) the intervention detailed the steps in a course of treatment or care in a plan, pathway, algorithm, guideline, protocol, or other ‘inventory of actions’ (i.e., the intervention had time-frames or criteria-based progression); and (iv) the intervention aimed to standardize care for a specific population. An intervention meeting all four criteria will be considered a CPW); (2) study was conducted in a hospital; (3) availability of information on implementation strategy(s) employed; (4) at least one health system outcome reported such as length of hospital stay in days, hospital costs, in-hospital mortality, adherence to recommended practice, and in-hospital complications. We will exclude studies on CPWs that are not focused on hospital interventions.

To ensure that the credibility and trustworthiness of the review are maintained, we will assess the rigor and relevance of each study’s potential contribution to the development of the program theory. Two independent reviewers will assess for rigor and relevance by examining whether the study inferences are based on evidence or author opinion. This reflexive process will use judgements and discussion to resolve conflicts. We will not exclude studies based on rigor. We will manage the screening and data extraction process using the Distiller SR software [35]. Where applicable, a third review member will resolve any disagreement during the screening process. Following a similar approach, we will assess gray literature and other sources of information relevant during the review process.

### Data extraction

Two review authors will independently extract data from all included primary studies using a piloted draft proposition sheet to extract explanatory account propositions (EAPs) about the program theory for CPWs implementation in hospitals. The EAPs are explanatory statements that may support, refine or refute judgements about an aspect or the program theory in its entirety [27]. Areas of data extraction will include the following:

- Study characteristics: title, author, publication year, publication status, country, participants, study focus (hospital interventions), hospital type, and hospital setting
- Intervention: relevance to the program theory, implementation strategies, and adoption support
- Program theory: explanatory accounts in CMO configuration, aspect(s) of program theory supported, refined or refuted, and other notes
- Methodology: relevance and study rigor

Relevance and rigor are dimensions of ‘fitness for purpose’ in realist synthesis. EAPs will be extracted from primary studies using an adapted format (“If-then-because”) for extracting propositions in realist inquiries [36]. If-then-because propositions assume that “outcome-*y*” will occur because “*z* mechanism” fire in context “*x*.” We will identify EAPs focused on the implementation and outcomes of CPWs in hospital settings. Subsequently, we will attempt to identify the CMOs from the extracted EAPs. Additionally, we will identify middle-range theories which are demi-regularities emerging from the data and are observable as a testable hypothesis to refine, support, or refute the program theory [27]. Where feasible, the extracted middle-range theories will be supported by formal theories identified from our ongoing systematic review update or other sectors apart from healthcare, where CPWs have been implemented. This step will be supported by the review teams’ theoretical and content expertise. All extracted data will be managed using Microsoft Excel. See (Additional file 2, part B) for the draft data extraction sheet to be used for the review.

### Data analysis and synthesis

Two review authors will extract the EAPs from the data extraction stage. Where necessary the team will consult with a review team to resolve any disagreement to improve the consistency and validity of the evidence generated. We will group the EAPs statements based on emerging themes, for example, “leadership support” or “staff empowerment.” Using abductive reasoning technique [37], we will identify the emerging CMOs configurations from thematic groupings of EAPs. Because the emerging themes from EAPs are not mutually exclusive, EAPs can be grouped into more than one thematic category. Where appropriate, we will juxtapose, reconcile, adjudicate, consolidate, and situate the evidence created within the framework of the program theory.

We will then identify the CMOs configuration existing within each thematic grouping and attempt to form CMOs configurations at the middle range theory level. Middle-range theories explain the regularities of social behavior that can be explored further by hypothesis testing [27]. The CMOs at the middle range theory level will be used to revise our initial program theory to form the initial revised program theory. All hidden and explicitly stated mechanisms [26] will be elicited with the support of content and theoretical expertise within the review team. Due to the focused scope of the review, we will only utilize key mechanisms in developing and refining the program theory; we will utilize mechanisms that yielded the largest impact on the outcomes of interest based on different contexts reported in the primary studies. This approach also makes the breadth and depth of the review manageable and tangible for translation and implementation. This process will be conducted in consultation with content specialists and other frontline users of CPWs in hospitals to refine the program theory. Since the quality assurance of our realist review is based on the reflexivity and explicitness of our review team and stakeholders involved, we cannot guarantee all fundamental mechanisms will be illuminated in the final program theory or that our results will be reproducible in other jurisdictions. Also, our literature search process may miss essential studies that may influence the final program theory. However, we will document and explain all changes conducted between the protocol and the realist review.

### Stakeholder consultation and refinement of the program theory

To ensure the relevance of the program theory, we will consult with a CPW stakeholder advisory group comprising of content experts, a realist methodologist, a patient representative, policy-makers, program managers, healthcare providers, and other CPWs frontline users. Content experts may include authors of primary studies focused on CPW implementation in hospitals or researchers who have successfully implemented CPWs in hospital settings. We will present the initial revised program theory and other study findings to the advisory group for their perspectives and to confirm, refine, or refute the initial revised program theory. The stakeholder engagement sessions will be delivered via facilitated discussion in a semi-structured format comprising of face-to-face and remote sessions to accommodate international participants. The engagement sessions will follow the guiding principles described by Manzano 2016 [38] for conducting approach for realist inquiry. The goal of the engagement sessions is to ensure the appropriateness of all aspects of the program theory and to make suggested changes where necessary. This process will further improve our understanding of the program theory for CPWs in hospital and ultimately lead to the development of the final revised program theory. We will develop a detailed knowledge translation plan with key decision-makers in Saskatchewan to facilitate the translation of the main findings to clinical practice.

### Ethics approval and consent to participate

Prior to consulting with stakeholders to help refine the draft program theory, ethical approval will be sought through the University of Saskatchewan Research Ethics Boards. All stakeholders will be consulted for consent to participate and publish.

## Discussion

Overall, the realist review aims to assess “how, why, to what extent and in what contexts does the effective implementation of clinical pathways in hospitals contribute to better health system outcomes?” As such, the review will create an evidence and theoretical base to support key decision-makers involved with the implementation of CPWs in hospitals. Building on the successful factors and key mechanisms of the final program theory with a detailed knowledge translation plan, we anticipate an improvement in the uptake of CPWs in hospitals. However, it is important to note that the steps described in this protocol will be conducted in an iterative manner, as opposed to a linear process which does not capture the complexities and iterations associated with realist reviews and complex interventions. The review will be disseminated via peer-reviewed journals, academic conferences, and as part of a graduate thesis. Finally, we anticipate an increase in beneficial effects from the future implementation of CPWs in hospitals, particularly those focused on the outcomes of interest in this review; length of hospital stay, costs, in-hospital mortality, adherence to recommended practice, and in-hospital complications.

## Additional files

Additional file 1: PRISMA-P 2015 Checklist. (DOCX 31 kb)

Additional file 2: Part A: MEDLINE Search Strategy. Search strategy conducted for on-going update of systematic review on the effects of clinical pathways in hospitals. Part B: draft data extraction sheet for realist review. (DOCX 39 kb)

## Data Availability

Data sharing is not applicable to this article as no datasets were generated or analyzed during this current study.

## Abbreviations

CMO: Context-mechanism-outcome
CPW: Clinical pathway
EAP: Explanatory account proposition
