# Supplementary material for: Development of a program theory for clinical pathways in hospitals: protocol for a realist review
Source: Syst Rev. 2019 Jun 8;8:136. doi: 10.1186/s13643-019-1046-0 (PMC6556023; doi:10.1186/s13643-019-1046-0)
Supplement: Supplementary file 2 — Part A: MEDLINE Search Strategy. Search strategy conducted for on-going update of systematic review on the effects of clinical pathways in hospitals. Part B: draft data extraction sheet for realist review. (DOCX 39 kb) [file 13643_2019_1046_MOESM2_ESM.docx]

**Part A: Medline Search Strategy.**

**Search strategy conducted for on-going update of systematic review on the effects of clinical pathways in hospitals.**

| Medline (OVID) |  |  |
| --- | --- | --- |
| Medline discs used: | Ovid MEDLINE(R) In-Process & Other Non-Indexed Citations and Ovid MEDLINE(R) 1946 to Present |  |
| 30-8-2016 | Epub Ahead of Print, In-Process & Other Non-Indexed Citations, Ovid MEDLINE(R) Daily and Ovid MEDLINE(R) 1946 to Present | Scroll down for search terms |
| 8-2-2017 | Ovid MEDLINE(R) Epub Ahead of Print, In-Process & Other Non-Indexed Citations, Ovid MEDLINE(R) Daily and Ovid MEDLINE(R) <1946 to Present> | Scroll down for search terms |
|  |  |  |
|  | Date searched (if different to summary sheet): |  |
|  |  |  |
| No. | Search terms | Results |
| 1 | Critical Pathways/ | 5140 |
| 2 | ((clinical or critical) adj2 (pathway? or path)).ti,ab. | 7456 |
| 3 | ((care adj2 algorithm?) or clinical algorithm?).ti,ab. | 1176 |
| 4 | (care adj2 pathway?).ti,ab. | 2616 |
| 5 | (treatment adj3 algorithm?).ti,ab. | 5539 |
| 6 | (structured care or intensive management).ti,ab. | 862 |
| 7 | (standardi$ adj3 (treatment? or care or patient care or plan$)).ti,ab. | 5862 |
| 8 | (care adj2 (plan? or map or maps or protocol? or algorithm?)).ti,ab. | 10333 |
| 9 | (protocol? adj4 (nursing or treatment or management or directed or guided)).ti,ab. | 25010 |
| 10 | ((local or locally) adj2 adapt$ adj5 guideline?).ti,ab. | 79 |
| 11 | (treatment model? adj10 standardi$).ti,ab. | 9 |
| 12 | (standardi$ adj3 (template or templates)).ti,ab. | 257 |
| 13 | or/1-12 [Pathways] | 59310 |
| 14 | Clinical protocols/ | 21974 |
| 15 | Algorithm/ and (di.fs. or (treatment or care or patient?).ti. or diagnos$.ti,ab.) | 44551 |
| 16 | Practice Guidelines as Topic/ or Guideline Adherence/ or Guidelines as topic/ | 136358 |
| 17 | ((guideline or guidelines) adj2 (adher$ or implement$)).ti,ab. | 5602 |
| 18 | (guideline? adj4 (compliance or complying)).ti,ab. | 2931 |
| 19 | or/16-18 [PGL or GL Adherence] | 139909 |
| 20 | (adherence or care or compliance or comply$ or implement$ or impact or plan? or standardi?ed or pathway or (treatment adj3 (protocol? or algorithm?))).ti,ab. | 2800181 |
| 21 | 19 and 20 [GL ] | 53817 |
| 22 | *Guidelines as topic/ or *Practice Guidelines as topic/ | 39468 |
| 23 | *Guideline Adherence/ | 11167 |
| 24 | or/22-23 [Focussed MeSH Guideline] | 47530 |
| 25 | Primary health care/ or Primary Care Nursing/ | 59553 |
| 26 | Family practice/ or General Practice/ | 66773 |
| 27 | General Practitioners/ or Physicians, Family/ or Physicians, Primary Care/ | 19441 |
| 28 | ((general or family) adj2 (practice? or practitioner? or physician? or doctor?)).ti,ab. | 97661 |
| 29 | (primary adj2 (care or health care or healthcare or medical care or patient care)).ti,ab. | 98355 |
| 30 | (primary care or family medic$ or general practice or family practi$).jn. | 8571 |
| 31 | GP.ti. | 3489 |
| 32 | or/25-31 [Primary Care ] | 232583 |
| 33 | Ambulatory Care/ or Community medicine/ or community health nursing/ or community health services/ or home care services/ or Community mental health services/ or Community Pharmacy Services/ | 127407 |
| 34 | Ambulatory Care Facilities/ or Community Health Centers/ | 19109 |
| 35 | (community or communities).ti,ab,hw. | 441147 |
| 36 | (((ambulatory or walk-in or neighbo?rhood or community) adj2 (clinic? or care centre or care centres or care center? or health$ centre or health$ centres or health$ center?)) or public clinic?).ti,ab. | 10485 |
| 37 | ((urban or rural) adj3 health).ti,ab. | 11119 |
| 38 | or/33-37 [Community Care] | 515822 |
| 39 | 13 and 32 [Pathway terms & PC] | 3083 |
| 40 | (and/13,38) not 39 [Pathways & Community-Ambulatory Care] | 3399 |
| 41 | (and/24,32) not (or/39-40) [Focussed GL & PC] | 3395 |
| 42 | (and/24,38) not (or/39-41) [Focussed GL & Community-Ambulatory Care] | 2247 |
| 43 | (21 and (or/32,38)) not (or/39-42) [GL & PC/Amb Care] | 6871 |
| 44 | ((or/14-15) and ((or/26-31,38) or *Primary health care/ or *Primary Care Nursing/)) not (or/39-43) [Clinical Protocols/Algorithms Mesh & PC/Community Care-combine with RCT filter only] | 3511 |
| 45 | (randomized controlled trial or controlled clinical trial).pt. or randomized.ab. or placebo.ab. or clinical trials as topic.sh. or randomly.ab. or trial.ti. | 1013198 |
| 46 | exp animals/ not humans.sh. | 4152952 |
| 47 | 45 not 46 [Cochrane RCT Filter 6.4.d Sens/Precision Maximizing] | 934229 |
| 48 | intervention?.ti. or (intervention? adj6 (clinician? or collaborat$ or community or complex or DESIGN$ or doctor? or educational or family doctor? or family physician? or family practitioner? or financial or GP or general practice? or hospital? or impact? or improv$ or individuali?e? or individuali?ing or interdisciplin$ or multicomponent or multi-component or multidisciplin$ or multi-disciplin$ or multifacet$ or multi-facet$ or multimodal$ or multi-modal$ or personali?e? or personali?ing or pharmacies or pharmacist? or pharmacy or physician? or practitioner? or prescrib$ or prescription? or primary care or professional$ or provider? or regulatory or regulatory or tailor$ or target$ or team$ or usual care)).ab. | 194948 |
| 49 | (pre-intervention? or preintervention? or "pre intervention?" or post-intervention? or postintervention? or "post intervention?").ti,ab. [added 2.4] | 13033 |
| 50 | (hospital$ or patient?).hw. and (study or studies or care or health$ or practitioner? or provider? or physician? or nurse? or nursing or doctor?).ti,hw. | 781323 |
| 51 | demonstration project?.ti,ab. | 2122 |
| 52 | (pre-post or "pre test$" or pretest$ or posttest$ or "post test$" or (pre adj5 post)).ti,ab. | 77801 |
| 53 | (pre-workshop or post-workshop or (before adj3 workshop) or (after adj3 workshop)).ti,ab. | 726 |
| 54 | trial.ti. or ((study adj3 aim?) or "our study").ab. | 760357 |
| 55 | (before adj10 (after or during)).ti,ab. | 394692 |
| 56 | ("quasi-experiment$" or quasiexperiment$ or "quasi random$" or quasirandom$ or "quasi control$" or quasicontrol$ or ((quasi$ or experimental) adj3 (method$ or study or trial or design$))).ti,ab,hw. | 114170 |
| 57 | ("time series" adj2 interrupt$).ti,ab,hw. | 1399 |
| 58 | (time points adj3 (over or multiple or three or four or five or six or seven or eight or nine or ten or eleven or twelve or month$ or hour? or day? or "more than")).ab. | 11310 |
| 59 | pilot.ti. | 46690 |
| 60 | Pilot projects/ | 92166 |
| 61 | (clinical trial or controlled clinical trial or multicenter study).pt. | 674918 |
| 62 | (multicentre or multicenter or multi-centre or multi-center).ti. | 34245 |
| 63 | random$.ti,ab. or controlled.ti. | 866032 |
| 64 | (control adj3 (area or cohort? or compare? or condition or design or group? or intervention? or participant? or study)).ab. not (controlled clinical trial or randomized controlled trial).pt. | 470246 |
| 65 | evaluation studies as topic/ or prospective studies/ or retrospective studies/ [Added Jan 2013] | 1079015 |
| 66 | (utili?ation or programme or programmes).ti. [Added Jan 2013] | 60368 |
| 67 | (during adj5 period).ti,ab. [Added Jan 2013] | 328905 |
| 68 | ((strategy or strategies) adj2 (improv$ or education$)).ti,ab. [Added Jan 2013] | 22433 |
| 69 | "comment on".cm. or review.pt. or (review not "peer review$").ti. or randomized controlled trial.pt. [Changed Jan 2013] | 3269864 |
| 70 | (rat or rats or cow or cows or chicken? or horse or horses or mice or mouse or bovine or animal?).ti. | 1420258 |
| 71 | exp animals/ not humans.sh. | 4152952 |
| 72 | (or/48-68) not (or/69-71) [EPOC Methods Filter 2.5-added Evaluation Studies line forward--Jan 20130 Medline] | 3177316 |
| 73 | (or/39-44) and 47 [RCT Results] | 3063 |
| 74 | (39 and 72) not 73 [EPOC Filter Results Set 1 : Pathways & PC] | 1293 |
| 75 | (40 and 72) not (or/73-74) [EPOC Filter Set 2: Pathways & Community-Ambulatory Care] | 1565 |
| 76 | (41 and 72) not (or/73-75) [EPOC Filter Set 3: Focussed GL & PC] | 1099 |
| 77 | (42 and 72) not (or/73-76) [EPOC Filter Set 4: Focussed GL & Ambultory] | 847 |
| 78 | (43 and 72) not (or/73-77) [EPOC Filter Set 5: GL & PC/Amb care] | 2586 |
| 79 | or/74-78 [EPOC Filter Results] | 7390 |
| 80 | 73 or 79 | 10453 |
| 81 | limit 80 to yr="2015 -Current" | 1257 |
|  |  |  |
|  |  |  |
| *2016 update: 30/08/2016* | |  |
|  |  |  |
| No. | Search terms | Results |
| 1 | Critical Pathways/ | 5420 |
| 2 | ((clinical or critical) adj2 (pathway? or path)).ti,ab. | 8083 |
| 3 | ((care adj2 algorithm?) or clinical algorithm?).ti,ab. | 1275 |
| 4 | (care adj2 pathway?).ti,ab. | 3099 |
| 5 | (treatment adj3 algorithm?).ti,ab. | 6141 |
| 6 | (structured care or intensive management).ti,ab. | 927 |
| 7 | (standardi$ adj3 (treatment? or care or patient care or plan$)).ti,ab. | 6438 |
| 8 | (care adj2 (plan? or map or maps or protocol? or algorithm?)).ti,ab. | 11126 |
| 9 | (protocol? adj4 (nursing or treatment or management or directed or guided)).ti,ab. | 26862 |
| 10 | ((local or locally) adj2 adapt$ adj5 guideline?).ti,ab. | 81 |
| 11 | (treatment model? adj10 standardi$).ti,ab. | 11 |
| 12 | (standardi$ adj3 (template or templates)).ti,ab. | 292 |
| 13 | or/1-12 [Pathways] | 64309 |
| 14 | Clinical protocols/ | 23218 |
| 15 | Algorithm/ and (di.fs. or (treatment or care or patient?).ti. or diagnos$.ti,ab.) | 46262 |
| 16 | Practice Guidelines as Topic/ or Guideline Adherence/ or Guidelines as topic/ | 142398 |
| 17 | ((guideline or guidelines) adj2 (adher$ or implement$)).ti,ab. | 6225 |
| 18 | (guideline? adj4 (compliance or complying)).ti,ab. | 3159 |
| 19 | or/16-18 [PGL or GL Adherence] | 146461 |
| 20 | (adherence or care or compliance or comply$ or implement$ or impact or plan? or standardi?ed or pathway or (treatment adj3 (protocol? or algorithm?))).ti,ab. | 3037238 |
| 21 | 19 and 20 [GL ] | 56974 |
| 22 | *Guidelines as topic/ or *Practice Guidelines as topic/ | 41843 |
| 23 | *Guideline Adherence/ | 11918 |
| 24 | or/22-23 [Focussed MeSH Guideline] | 50500 |
| 25 | Primary health care/ or Primary Care Nursing/ | 62173 |
| 26 | Family practice/ or General Practice/ | 69506 |
| 27 | General Practitioners/ or Physicians, Family/ or Physicians, Primary Care/ | 22382 |
| 28 | ((general or family) adj2 (practice? or practitioner? or physician? or doctor?)).ti,ab. | 101817 |
| 29 | (primary adj2 (care or health care or healthcare or medical care or patient care)).ti,ab. | 106166 |
| 30 | (primary care or family medic$ or general practice or family practi$).jn. | 8827 |
| 31 | GP.ti. | 3764 |
| 32 | or/25-31 [Primary Care ] | 244482 |
| 33 | Ambulatory Care/ or Community medicine/ or community health nursing/ or community health services/ or home care services/ or Community mental health services/ or Community Pharmacy Services/ | 130322 |
| 34 | Ambulatory Care Facilities/ or Community Health Centers/ | 22206 |
| 35 | (community or communities).ti,ab,hw. | 476877 |
| 36 | (((ambulatory or walk-in or neighbo?rhood or community) adj2 (clinic? or care centre or care centres or care center? or health$ centre or health$ centres or health$ center?)) or public clinic?).ti,ab. | 11292 |
| 37 | ((urban or rural) adj3 health).ti,ab. | 12016 |
| 38 | or/33-37 [Community Care] | 556500 |
| 39 | 13 and 32 [Pathway terms & PC] | 3382 |
| 40 | (and/13,38) not 39 [Pathways & Community-Ambulatory Care] | 3691 |
| 41 | (and/24,32) not (or/39-40) [Focussed GL & PC] | 3562 |
| 42 | (and/24,38) not (or/39-41) [Focussed GL & Community-Ambulatory Care] | 2399 |
| 43 | (21 and (or/32,38)) not (or/39-42) [GL & PC/Amb Care] | 7218 |
| 44 | ((or/14-15) and ((or/26-31,38) or *Primary health care/ or *Primary Care Nursing/)) not (or/39-43) [Clinical Protocols/Algorithms Mesh & PC/Community Care-combine with RCT filter only] | 3722 |
| 45 | (randomized controlled trial or controlled clinical trial).pt. or randomized.ab. or placebo.ab. or clinical trials as topic.sh. or randomly.ab. or trial.ti. | 1063538 |
| 46 | exp animals/ not humans.sh. | 4304227 |
| 47 | 45 not 46 [Cochrane RCT Filter 6.4.d Sens/Precision Maximizing] | 980712 |
| 48 | intervention?.ti. or (intervention? adj6 (clinician? or collaborat$ or community or complex or DESIGN$ or doctor? or educational or family doctor? or family physician? or family practitioner? or financial or GP or general practice? or hospital? or impact? or improv$ or individuali?e? or individuali?ing or interdisciplin$ or multicomponent or multi-component or multidisciplin$ or multi-disciplin$ or multifacet$ or multi-facet$ or multimodal$ or multi-modal$ or personali?e? or personali?ing or pharmacies or pharmacist? or pharmacy or physician? or practitioner? or prescrib$ or prescription? or primary care or professional$ or provider? or regulatory or regulatory or tailor$ or target$ or team$ or usual care)).ab. | 218683 |
| 49 | (pre-intervention? or preintervention? or "pre intervention?" or post-intervention? or postintervention? or "post intervention?").ti,ab. [added 2.4] | 15304 |
| 50 | (hospital$ or patient?).hw. and (study or studies or care or health$ or practitioner? or provider? or physician? or nurse? or nursing or doctor?).ti,hw. | 810807 |
| 51 | demonstration project?.ti,ab. | 2216 |
| 52 | (pre-post or "pre test$" or pretest$ or posttest$ or "post test$" or (pre adj5 post)).ti,ab. | 86771 |
| 53 | (pre-workshop or post-workshop or (before adj3 workshop) or (after adj3 workshop)).ti,ab. | 820 |
| 54 | trial.ti. or ((study adj3 aim?) or "our study").ab. | 842176 |
| 55 | (before adj10 (after or during)).ti,ab. | 416033 |
| 56 | ("quasi-experiment$" or quasiexperiment$ or "quasi random$" or quasirandom$ or "quasi control$" or quasicontrol$ or ((quasi$ or experimental) adj3 (method$ or study or trial or design$))).ti,ab,hw. | 124033 |
| 57 | ("time series" adj2 interrupt$).ti,ab,hw. | 1728 |
| 58 | (time points adj3 (over or multiple or three or four or five or six or seven or eight or nine or ten or eleven or twelve or month$ or hour? or day? or "more than")).ab. | 12735 |
| 59 | pilot.ti. | 51666 |
| 60 | Pilot projects/ | 96615 |
| 61 | (clinical trial or controlled clinical trial or multicenter study).pt. | 682423 |
| 62 | (multicentre or multicenter or multi-centre or multi-center).ti. | 37580 |
| 63 | random$.ti,ab. or controlled.ti. | 934497 |
| 64 | (control adj3 (area or cohort? or compare? or condition or design or group? or intervention? or participant? or study)).ab. not (controlled clinical trial or randomized controlled trial).pt. | 507163 |
| 65 | evaluation studies as topic/ or prospective studies/ or retrospective studies/ [Added Jan 2013] | 1127398 |
| 66 | (utili?ation or programme or programmes).ti. [Added Jan 2013] | 64495 |
| 67 | (during adj5 period).ti,ab. [Added Jan 2013] | 345785 |
| 68 | ((strategy or strategies) adj2 (improv$ or education$)).ti,ab. [Added Jan 2013] | 25129 |
| 69 | "comment on".cm. or review.pt. or (review not "peer review$").ti. or randomized controlled trial.pt. [Changed Jan 2013] | 3408192 |
| 70 | (rat or rats or cow or cows or chicken? or horse or horses or mice or mouse or bovine or animal?).ti. | 1476130 |
| 71 | exp animals/ not humans.sh. | 4304227 |
| 72 | (or/48-68) not (or/69-71) [EPOC Methods Filter 2.5-added Evaluation Studies line forward--Jan 20130 Medline] | 3387408 |
| 73 | (or/39-44) and 47 [RCT Results] | 3303 |
| 74 | (39 and 72) not 73 [EPOC Filter Results Set 1 : Pathways & PC] | 1418 |
| 75 | (40 and 72) not (or/73-74) [EPOC Filter Set 2: Pathways & Community-Ambulatory Care] | 1681 |
| 76 | (41 and 72) not (or/73-75) [EPOC Filter Set 3: Focussed GL & PC] | 1167 |
| 77 | (42 and 72) not (or/73-76) [EPOC Filter Set 4: Focussed GL & Ambultory] | 902 |
| 78 | (43 and 72) not (or/73-77) [EPOC Filter Set 5: GL & PC/Amb care] | 2716 |
| 79 | or/74-78 [EPOC Filter Results] | 7884 |
| 80 | 73 or 79 | 11187 |
| 81 | limit 80 to yr="2015 -Current" | 1138 |
|  |  |  |
| *2017 update: 08/02/2017* | |  |
|  |  |  |
|  |  |  |
| No. | Search terms | Results |
| 1 | (clinical adj2 pathway?).ti. | 1285 |
| 2 | critical pathways/ | 5562 |
| 3 | ((clinical or critical) adj1 (pathway? or path?)).ti,ab. | 5259 |
| 4 | ((care adj2 algorithm?) or clinical algorithm?).ti,ab. | 1315 |
| 5 | (care adj1 pathway?).ti,ab. | 2703 |
| 6 | (treatment adj3 algorithm?).ti,ab. | 6455 |
| 7 | (management protocol? or treatment protocol?).ti,ab. | 17810 |
| 8 | (care adj1 (plan? or map?)).ti,ab. | 7232 |
| 9 | (protocol? adj1 (nursing or directed or guided)).ti,ab. | 547 |
| 10 | ((local or locally) adj2 adapt* adj5 guideline?).ti,ab. | 85 |
| 11 | (treatment model? adj10 standardi*).ti,ab. | 11 |
| 12 | (standardi* adj3 protocol?).ti,ab. | 9894 |
| 13 | systematic detection.ti,ab. | 271 |
| 14 | or/2-13 | 53386 |
| 15 | clinical protocols/ | 24668 |
| 16 | (treat* or therap*).ti,ab. | 5628132 |
| 17 | 15 and 16 | 12241 |
| 18 | practice guidelines as topic/ | 97006 |
| 19 | (implement* or pathway or protocol?).ti,ab. | 1185547 |
| 20 | 18 and 19 | 12971 |
| 21 | (guideline? adj1 (implement* or pathway or protocol?)).ti,ab. | 2112 |
| 22 | or/20-21 | 14130 |
| 23 | 14 or 17 or 22 | 76751 |
| 24 | (hospital or hospitals or hospitalis* or hospitaliz*).ti,ab. | 1022715 |
| 25 | exp hospital units/ | 90944 |
| 26 | exp hospitals/ | 241004 |
| 27 | exp hospital departments/ | 161398 |
| 28 | hospitalization/ | 85875 |
| 29 | or/24-28 | 1287170 |
| 30 | 1 or (23 and 29) | 18164 |
| 31 | randomized controlled trial.pt. | 447592 |
| 32 | controlled clinical trial.pt. | 91833 |
| 33 | multicenter study.pt. | 218061 |
| 34 | pragmatic clinical trial.pt. | 521 |
| 35 | (randomis* or randomiz* or randomly).ti,ab. | 723337 |
| 36 | groups.ab. | 1672872 |
| 37 | (trial or multicenter or multi center or multicentre or multi centre).ti. | 204098 |
| 38 | (intervention? or effect? or impact? or controlled or control group? or (before adj5 after) or (pre adj5 post) or ((pretest or pre test) and (posttest or post test)) or quasiexperiment* or quasi experiment* or pseudo experiment* or pseudoexperiment* or evaluat* or time series or time point? or repeated measur*).ti,ab. | 7883302 |
| 39 | non-randomized controlled trials as topic/ | 122 |
| 40 | interrupted time series analysis/ | 239 |
| 41 | controlled before-after studies/ | 214 |
| 42 | or/31-41 | 8810908 |
| 43 | exp animals/ | 20711674 |
| 44 | humans/ | 16396415 |
| 45 | 43 not (43 and 44) | 4315259 |
| 46 | review.pt. | 2223092 |
| 47 | meta analysis.pt. | 73858 |
| 48 | news.pt. | 180989 |
| 49 | comment.pt. | 678358 |
| 50 | editorial.pt. | 425203 |
| 51 | cochrane database of systematic reviews.jn. | 12923 |
| 52 | comment on.cm. | 678357 |
| 53 | (systematic review or literature review).ti. | 90278 |
| 54 | or/45-53 | 7523264 |
| 55 | 42 not 54 | 6145029 |
| 56 | 30 and 55 | 10214 |
| 57 | limit 56 to yr="2008 -Current" | 6218 |

**Part B: Draft data extraction sheet for realist review**

| Data extraction sheet | | Funding source | |
| --- | --- | --- | --- |
| Date | | Reviewer’s Initial | |
| Hospital type and section | |  | |
| Author and year | | | |
| Country | | | |
| Participants | | | |
| Implementation strategy(s) | | | |
| For what aspect(s) of program theory does this study provide evidence (i.e. evidence ‘to support, refute or refine elements of theory)? *(Include a summary of the nature of the evidence and quotes/ page numbers if possible).* | | | |
| CMO Table (Or elements of CMOs) (Add extra lines if required). Include quotes or page no’s | | | |
| Context | Mechanism | | Outcome |
|  |  | |  |
|  |  | |  |
|  |  | |  |
| What amendments to program theory might you propose based on this study? | | | |
| Comments or questions on rigour/methodology | | | |
| Priority for in-depth review: Please rank as High / Medium / Low.  Why? | | | |
| Other notes/comments | | | |
